# Identification of factors required for meristem function in Arabidopsis using a novel next generation sequencing fast forward genetics approach

**DOI:** 10.1186/1471-2164-12-256

**Published:** 2011-05-20

**Authors:** Michal Mokry, Isaäc J Nijman, Anja van Dijken, Rene Benjamins, Renze Heidstra, Ben Scheres, Edwin Cuppen

**Affiliations:** 1 Hubrecht Institute, Developmental Biology and Stem Cell Research, KNAW and University Medical Center Utrecht, Uppsalalaan 8, Utrecht, The Netherlands; 2 Utrecht University, Faculty of Science, Department of Biology, Section Molecular Genetics, Padualaan 8, Utrecht, The Netherlands; 3 Department of Medical Genetics, University Medical Center Utrecht, Universiteitsweg 100, The Netherlands

## Abstract

**Background:**

Phenotype-driven forward genetic experiments are powerful approaches for linking phenotypes to genomic elements but they still involve a laborious positional cloning process. Although sequencing of complete genomes now becomes available, discriminating causal mutations from the enormous amounts of background variation remains a major challenge.

**Method:**

To improve this, we developed a universal two-step approach, named 'fast forward genetics', which combines traditional bulk segregant techniques with targeted genomic enrichment and next-generation sequencing technology

**Results:**

As a proof of principle we successfully applied this approach to two Arabidopsis mutants and identified a novel factor required for stem cell activity.

**Conclusion:**

We demonstrated that the 'fast forward genetics' procedure efficiently identifies a small number of testable candidate mutations. As the approach is independent of genome size, it can be applied to any model system of interest. Furthermore, we show that experiments can be multiplexed and easily scaled for the identification of multiple individual mutants in a single sequencing run.

## Background

Since the groundbreaking work of William Bateson and Thomas H. Morgan at the beginning of last century describing the key concepts of genetic linkage and linearly ordered genes on chromosomes, phenotype-driven forward genetics has developed into one of the most powerful approaches for assigning genomic elements to biological function. Many novel biological concepts have been discovered using this approach, including human disease genes like Duchenne Muscular Dystrophy [1], Huntington's [2] and Cystic Fibrosis [3] but also completely novel classes of biomolecules like microRNAs were identified this way [4,5]. While many studies depended on naturally occurring mutations, the use of radiation and chemical mutagens dramatically boosted the generation of phenotypic mutants and eventually led to the first saturation screens allowing for the systematic identification of genes involved in specific processes [6].

Despite these successes, the process of positional cloning, i.e. the identification of the gene and variant(s) that causes the phenotype has remained a challenging and laborious process. Although it took until the 1980's before the gene underlying one of the most studied mutants, the Drosophila white-eye phenotype that was studied by Morgan in the 1910's, was identified [7], versatile techniques have since been developed to facilitate the cloning process. While tag-based mutagenesis approaches using e.g. transposons [8,9] or viruses [10] facilitated the cloning process, chemical-based methods using e.g. EMS or ENU [11-13] have remained most popular because of their highly random distribution and high efficiency. With the advent of next-generation sequencing technologies [14], it now has become possible to sequence complete genomes of individual mutants, thereby providing comprehensive inventories of genetic variation. However, it has also become clear that this information alone is not sufficient for unequivocally linking a phenotype to a specific genotype or single mutation. In genetically heterogeneous populations, large amounts of background variation is present, including hundreds of apparently deleterious mutations like premature stop codons in protein-coding genes [15,16]. Similar problems arise in model organisms; since chemical mutagenesis typically results in many thousands of induced mutations per genome. Hence, discriminating background variation from causal mutations remains a major challenge. Indeed, a recent study in *C. elegans *demonstrated that even with the power of next-generation sequencing technologies and relatively small genome size, additional linkage information is required for mutation cloning [17]. Although such data can be obtained by classical genetic mapping experiments or, alternatively, background variation could be eliminated by extensive backcrossing, these are relatively laborious processes. To address this, we developed a two-step protocol, named 'fast forward genetics', that combines a traditional bulk-segregant analysis approach [18] with state-of-the-art next-generation sequencing techniques [19]. The fast forward genetics approach is highly efficient compared to traditional approaches and while we provide proof-of-principle using *Arabidopsis thaliana *it can in principle be applied to any sequenced species of interest and is largely independent of the genome size. Furthermore, we show that multiple samples can be processed simultaneously using multiplexed barcoded/indexed samples in a single sequencing run.

## Results

### Fast forward genetics principle

**Figure 1:**
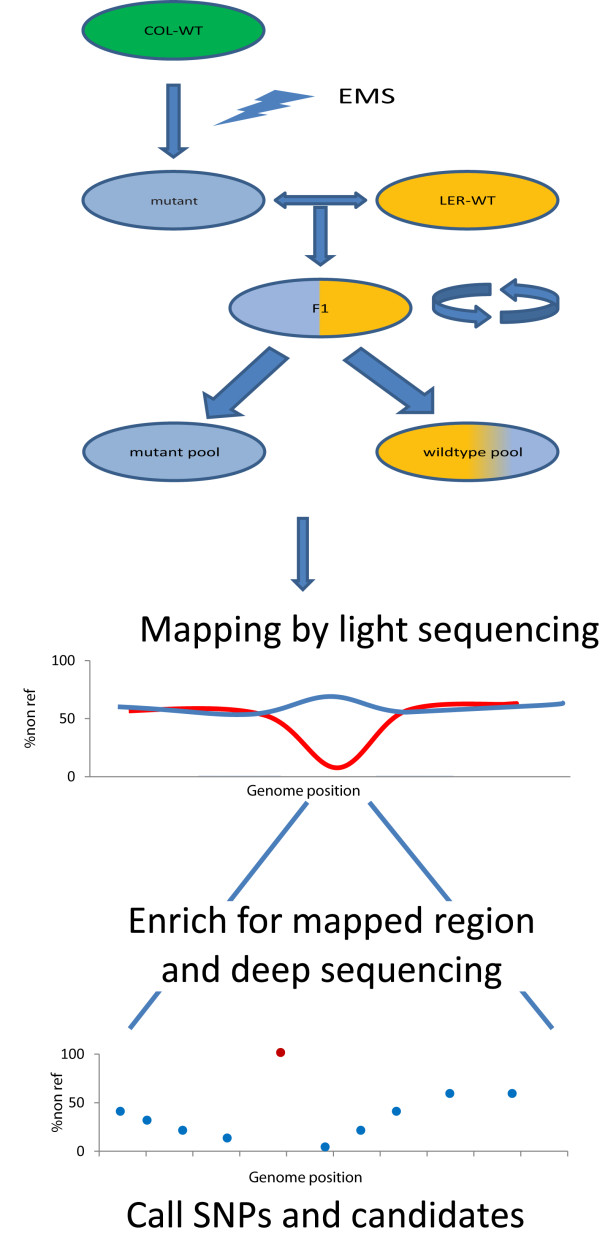
**Schematic representation of the two step fast forward genetics approach**. The mutant ecotype is crossed to a mapping ecotype background. The resulting F1 progeny are subsequently selfed to generate a F2 population in which the mutant phenotype segregates according to Mendelian rules (25% mutant, 75% wild type). Equal numbers of 50 to 200 mutant and wild type individuals are pooled (bulk segregant pools) and used in the procedure. First, 'light sequencing (1 to 10 × genome coverage) is used to map the mutation to a genomic region, which is revealed by a strong decrease of mapping ecotype alleles in the mutant pool and a mild increase in the wild type pool. Next, a capture array is designed to capture DNA from the linked region in the mutant pool, followed by deep sequencing. This results in the identification of low frequency SNP alleles from the mapping ecotype, which allow for fine-mapping as well as abundant (homozygotic) novel non-reference alleles (red dot). These variants are prime candidates for the mutation causing the phenotype since it is the only common variant in all the individuals in the mutant pool.

First, bulk-segregant mutant and wild-type pools are generated by outcrossing the mutant ecotype to a polymorphic mapping ecotype, followed by selfing of the F1 progeny to establish the F2 generation in which the recessive phenotype segregates in a Mendelian fashion (Figure 1). From these, pools of mutant as well as wild type segregants were generated. While in the first pool, the causal mutation will be homozygous, the wild-type pool contains both heterozygous mutant as well as homozygous wild type individuals. Polymorphic alleles introduced by the mapping ecotype background, however, function as genetic markers and will be randomly distributed in both pools and appear at a frequency of ~50%, unless the marker is linked to the causal mutation. In such cases, the non-reference mapping allele will decrease gradually towards 0% in the mutant pool upon getting closer to the causal variant, while increasing to 67% in the wild-type pool (Figure 1). Traditionally, hundreds of genetic markers (e.g. RFLPs, AFLPs, SNPs [20-22]) that are evenly distributed over the genome are tested in simple or multiplexed assays to roughly map the mutation to a chromosome or chromosomal segment. This step is normally followed by a fine-mapping step in non-pooled F2 individuals and requires additional markers in the region of interest. Finally, candidate genes are sequenced to identify the causal mutation.

In the fast forward genetics approach, all these steps are combined in a two-step experimental approach (Figure 1). When large sets of known SNPs are available (e.g. when complete genome sequences are available), the mutant pool can be 'lightly' sequenced using short read next-generation sequencing to obtain linkage information (Figure 1). Although high nucleotide coverage (>20 ×) is typically required for reliable genotyping by next-generation sequencing, the availability of high-density SNP panels for most commonly used model organism systems, makes it possible to use low sequence coverage (1 - 2 ×) to generate medium resolution linkage information using bins of markers. Per bin of for example 100 known SNP positions, all reads covering a known polymorphic position are collected and the ratio between raw reads representing reference alleles and mapping alleles is calculated. Interestingly, this step makes the approach largely independent of the genome size. The size of the bin can be adapted to the depth of sequencing, the number of markers available and the size of the genome. While, as a consequence, the mapping resolution might be variable, it should be noted that mapping resolution depends on the number of meiotic crossovers and is thus largely determined by the size of the bulk-segregant pools.

Alternatively, when no high-density SNP data is available, the wild type pool can be sequenced at low coverage in addition to the mutant pool. The combined data can first be used to reveal variable positions between the ecotypes used. Next, these positions are typed for both pools separately and again ratios between reference and mapping alleles are calculated (Figure 1). In the study described here we applied the de-novo identification and typing of variants in the population for mapping.

After the initial mapping step, which typically results in linkage to a 5 to 10 centiMorgan region, a capture array is designed (custom 1 M or 244 k Agilent SurePrint) to enrich for DNA fragments of the linked genomic region (typically 1 to 10 Mbp). In the second step the mutant bulk-segregant pool is enriched and deep-sequenced to about 5 to 10 × per allele in the pool (~1,000 × in total). This allows for simultaneous fine-mapping (step-wise decrease of mapping allele frequency due to individual cross-over events) and candidate mutation identification (fully homozygous non-reference alleles) (Figure 1).

### Bulk-segregant analysis by 'light sequencing'

For a proof-of-principle, we selected two novel recessive Arabidopsis mutants. The *picup1 *and *twirt1 *(*twr-1*) mutants display altered root meristem function resulting in short roots, with *twr-1 *mutants also affecting the shoot meristem. The mutants were generated by chemical EMS-mutagenesis of transgenic marker lines. By crossing to the Ler-1 ecotype a mapping population was generated from which the mutant as well as the wild type/heterozygous pools 200 plants each, were generated. Subsequently, DNA of the corresponding pools was converted into standard fragment libraries and sequenced in multiplex setup using barcodes that were introduced during library preparation. Next-generation sequencing was performed using AB/SOLiD technology and because of the relatively small size of the Arabidopsis genome (~120 Mbp) even the use of only about 10% of the current capacity of a single slide run results in on average 10 × genome coverage (Table 1). Sequence reads were mapped to the TAIR 8 reference genome and variable positions common to the mutant and wild-type pool are used for mapping. The mutant/mapping allele (Col/Ler) ratio was calculated for bins of 25 mapping SNPs. Simulations using part of the sequencing data showed that the linkage results are similar for even lower depth sequencing down to ~1 × coverage (Additional file 1, Figure S1A). Below this coverage, SNP discovery becomes too unreliable and a known SNP set should be used. The number of SNPs per bin can also be varied (Additional file 1, Figure S1B). A higher number results in a smoother curve, but this is only possible when sufficient SNPs are available or discovered. When this number is limited, the effect of single SNPs becomes more pronounced and can cause large fluctuations in the frequency for a bin.

**Table 1:** Sequencing statistics for Arabidopsis mutants using fast forward genetics

Mutant	pool	raw reads	mapped reads	on target reads (%)	Avg coverage (genome/*target*)
	mutant	33,153,386	24,617,730		10.3×
*picup1*	wild type	19,696,216	14,776,790		6.2×
	mutant enriched	51,643,460	40,185,359	35,541,544 (88%)	*1,777*×

	mutant	29,766,024	23,150,679		9.6×
*twr-1*	wild type	20,687,086	16,308,193		6.8×
	mutant enriched	46,068,250	37,584,650	32,882,209 (87%)	*1,644*×

**Figure 2:**
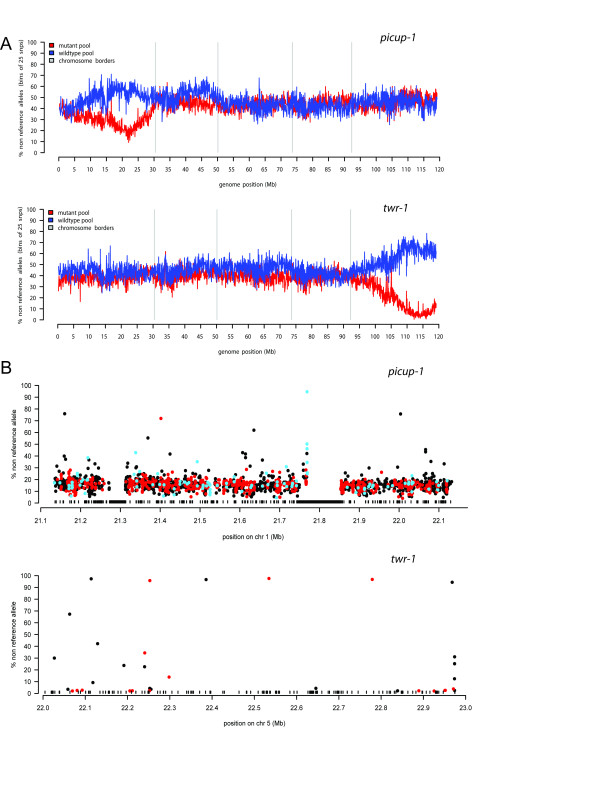
**Results of the fast forward genetics approach.** A) First step of the fast forward genetics approach. Single linkage to genomic region was identified for both mutants. The region with low frequency non-reference alleles in the mutant population indicates a common genomic fragment to the mutant pool. Verification in the wild type pool (blue graph) supports this region by showing an overrepresentation of non-reference alleles to balance the Hardy-Weinberg equilibrium. B) Second step of the fast forward genetics approach. Non-reference alleles detected in the genomic enrichment sequencing data are plotted by their location and frequency for mutants (black: non-coding, red: coding, blue: UTR). The black boxes indicate regions where no capture probes could be designed due to repeats and/or other non-unique sequences

A single linkage region was identified on chromosome 1 around 21.6 Mb for *picup-1 *and on chromosome 5 around 22.5 Mb (Figure 2A) for *twr-1 *mutant. Analysis of the wild-type pool supported these locations as the percentage of non-reference alleles increases at the same locations. In addition, the mutants were analyzed in a traditional mapping approach using a panel of PCR-based markers (not shown). The results of that experiment were fully consistent with the fast forward genetics analysis as the *picup-1 *mutation on chromosome 1 between 21.6 and 21.9 Mb spanning 285 kb and the *twirt *mutation was mapped between 22.4 and 22.6 Mb on chromosome 5 spanning a 212 kb region (data not shown).

### Genomic enrichment and 'deep-sequencing'

Recently, various techniques have been developed for the enrichment of specific genomic DNA fragments for down-stream next-generation sequencing [23-26]. Although in principle many different platforms could be used for this step, we have chosen to use custom-designed Agilent microarrays to enrich the bulk segregant libraries, as this platform is highly flexible in terms of design and ordering. Furthermore, in combination with short-insert fragment libraries and AB/SOLiD sequencing, these arrays provide high target-enrichment rates with relatively even coverage, which was found to be instrumental for reliable heterozygous mutation discovery with high sensitivity and specificity [27,28].

A single microarray with a dense tiling set of probes targeting 1 Mb of the (repeat-masked) region identified in the mapping was designed and used for the enrichment of the mutant pool. The enriched sample was sequenced using AB/SOLiD, in barcoded/multiplex way using ~ 10-15% of the capacity of a single slide run. The enrichment was highly efficient with 87-88% of the reads mapping to the target region of interest, resulting in 1,777 × coverage for the *picup1 *mutant and 1,644 × coverage for the *twr-1 *mutant (~ 4.1-4.4 × per allele in the pool) (Table 1).

To identify potential causal variants, we calculated the percentage of non-reference alleles for every informative position in the regions of interest. This percentage is expected to decrease for polymorphic mapping ecotype alleles when closing in to the causal variant and potential causal variants are expected to show up as 100% non-reference. To account for noise (error rates in sequencing as well as mapability issues) in the raw sequencing data as well as potential missorting of individuals in the mutant pool, relatively loose criteria (>70% non-reference alleles) were applied for candidate mutations.

Using these settings, for the *picup1 *mutant, four variants were found in targeted region, two in non-coding regions, one silent and one candidate identified in the 3'-UTR of At1g58602. Although these variants could be causal mutations, the mapped region also contains a sub-region with highly repetitive sequences that was omitted from the capture design (Figure 2B). Furthermore, in this mutant 95.15% of targeted sequence was covered at more than 20x, leaving 13,391 coding bases non-inspected. A large proportion of these were in the two obvious gaps (Figure 2B) which were excluded from the array design criteria due to known repeat content or sequences present multiple times in the genome (duplications). The first gap around 21.3 Mb contains a large cluster of non-coding RNA genes and does not contain CDS regions, but the second gap around 21.8 Mb contains large transposable elements and coding sequences with high similarity matches elsewhere in the genome (some of them to mitochondrial and chloroplast). Because of their annotation as repeats (TAIR8 built) these sequences were automatically omitted from the design of the enrichment array since they would either decrease capture efficiency or introduce difficulties in mapping the sequences back to the genome.

**Figure 3:**
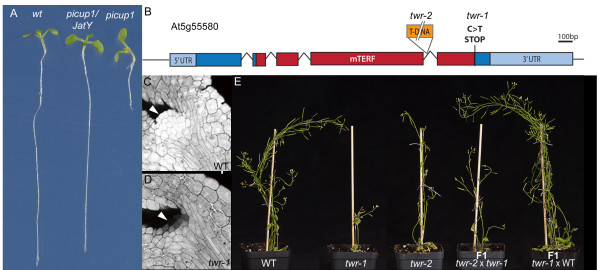
**Identification and characterization of the mutants**. A) Complementation of the *picup1 *mutant by one of the JatY clones. B) Schematic representation of the TWR gene. Boxes indicate coding sequence. The conserved mTERF domain (pfam PF02536) in red, UTRs in light blue. *twr-1*: point mutation C > T causing premature stop-codon at the end of the mTERF domain. *twr-2*: T-DNA insertion in intron 4. C, D) Aniline blue staining of mature embryos showing a dome shaped group of cells in wt representing the shoot apical meristem (C), which appears absent in the *twr-1 *mutant embryo (D). E) Complementation analysis of the cross between *twr-1 *and *twr-2 *shows identical above ground phenotypes to the single mutants. A backcross of *twr-1 *to wt phenotypically resembles the wt.

For *twr-1 *only a very limited number of candidate mutations were identified (Figure 2B, Table 2). As expected, the majority (5 out of 6) variants are G/C to A/T mutations, which is in line with the EMS-induced mutation spectrum in Arabidopsis [29]. We predicted the effect of the candidates (Table 2), and we found a candidate causing a premature stop codon in the gene At5g55580 (Figure 3A) making this a very likely cause for the phenotype. When we consider the additional PCR-based mapping information, it is the only candidate in the region.

**Table 2:** Detected candidate mutants and their predicted effect on the genes (green: silent, yellow: synonymous, red: stop).

mutant	Chr	Position (TAIR8)	ref allele	Detected allele	gene	Predicted effect
	chr1	21,160,008	C	T	AT1G56490	pseudogene
*picup-1*	chr1	21,401,749	G	A	AT1G57770	S558S
	chr1	21,768,825	G	A	AT1G58602	3'UTR
	chr1	22,003,621	T	C		

	chr5	22,113,982	T	A		
	chr5	22,252,705	C	T	AT5G54730	A345T
*twr-1*	chr5	22,385,804	C	T		
	chr5	22,534,542	C	T	AT5G55580	Q467X
	chr5	22,779,060	C	T	AT5G56240	M405I
	chr5	22,968,138	C	T		

On the other hand, it cannot completely be excluded that the causal mutations were missed by sequencing and are still presented in targeted regions. However, sequencing the target region for mutant showed that 99.86% of all coding bases were covered over 20x, which is sufficient for our strict SNP calling pipeline, and only 471 coding bases were not efficiently surveyed.

### Mutant phenotypes and complementation test

The *picup1 *mutant displays mis-localization of the normally polar localized PIN2 auxin efflux facilitator, which results in a shorter root (Figure 3E). To confirm that the causal mutation in the *picup1 *mutant is residing within the identified repetitive region we ordered JatY clones (http://www.jicgenomelab.co.uk) spanning this region and used these to transform two different *picup1 *alleles that came from the same mutant screen and checked for complementation in the F2 generation. Four of the JatY clones were able to complement both mutant alleles (Additional file 1, Figure S2). While these results confirm the mapping data, identification and confirmation of the causal variant will require additional experiments, which are complicated by the repetitive nature of this region and potential incompleteness of this region in the reference genome.

The *twr-1 *mutant shows a reduced root growth and delayed shoot meristem activation resulting in belated outgrowth of leaves. The shoot defect was traced back to the embryo development with mature mutant embryos displaying a largely reduced shoot apical meristem compared to the dome shaped meristem in the WT (Figure 3B,C). Upon transition to flowering the plant produces fewer stems and enhanced floral termination resulting in reduced seed set (Figure 3D).

To confirm that the causal mutation in the *twr-1 *mutant maps to the *At5g55580 *gene encoding a mitochondrial transcription termination factor (mTERF) family protein, we obtained a second allele harboring a T-DNA insertion in the fourth intron. This allele displayed phenotypes very similar to *twr-1 *and subsequent complementation analysis revealed they are allelic (Figure 3D). Accordingly we renamed this mutant allele *twr-2*.

## Discussion

We show that with the current sequencing technologies and DNA capture approaches unknown mutations can be mapped robustly to a single genomic region. Furthermore, a testable number of causal candidate mutations can routinely be obtained in a very fast and scalable two-step approach. In addition, multiple mutants can be analyzed simultaneously using a multiplexed setup with individually barcoded samples in single sequencing run. While the current capacity of next-generation sequencing equipment would already allow for the analysis of 10 to 20 Arabidopsis mutants in a single run, it is clear that the generation of mutants, bulk-segregant pools and mainly down-stream validation experiments will soon become the limiting step in forward genetics. While validation can be very laborious, the increased efficiency of the mapping and cloning steps opens up opportunities to pursue the identification of multiple independent alleles for the same locus.

The key feature of the fast forward genetics approach is the use of an old genetic trick, the use of cumulative information from bulk segregant pools. Theoretically, a pool of 100 individuals contains the information required for mapping to a resolution of about 1 cM. Translation into physical size depends on the species and chromosomal region. In general, however, a 1 cM region will typically not be larger than 1-5 Mb. While increasing pool sizes above 100 might increase mapping resolution, this would also require more sequencing depth. More importantly, however, sequencing error rates need to be significantly lower than the single allele frequency to be able to detect single cross-over events. When 500 individuals are pooled, a sequencing accuracy of 99,9% (as currently claimed by most platforms) would mean that single allele signals equal background signal and would not be distinguishable.

Based on the known EMS-induced mutation frequency of about 1 in 100 kb [29], the regions resulting from the mapping step are expected to contain ~10 candidate mutations. We find only slightly lower numbers (four to six), which is probably due to a less efficient EMS treatment in these experiments and/or the fact that we did not efficiently cover repetitive regions. Nevertheless, fine-mapping nucleotide resolution deep-sequencing as well as bioinformatic analysis resulted in a very limited number of candidate mutations that are testable in functional assays like rescue by large-insert genomic clones or analysis of independent insertion lines from public resources (e.g. http://signal.salk.edu).

While one could in principle perform the low-resolution mapping step using traditional methods, followed by enrichment and sequencing of the non-pooled mutant ecotype genome for the linkage region, with decreasing costs of consumables, the possibility of multiplexing and increasing throughput of next generation sequencing platforms, the costs of low resolution mapping step using deep sequencing became comparable or superior to traditional methods. In any case, only a single next-generation sequencing fragment library has to be prepared and sequenced for the whole two-step procedure.

Although not strictly required for the fast forward genetics approach, the wild-type pool can be included in experiments can have advantages. First, they function as controls for the identification of real linkage locations in the mutant pools as an increase of mapping ecotype allele frequencies are expected at the same chromosomal location in the wild-type pools. Secondly, combining mutant and wild-type sequencing data allows for de novo calling of SNPs, which might be useful when no or incomplete SNP inventories are available for the ecotype combination used. SNP discovery criteria can be set fairly stringent, as only several thousand markers genome-wide are required for the mapping step. Thirdly, sequencing data from the wild-type pool allows for the filtering of apparent candidate mutations in the second step that are actually due to genetic differences between the ecotype used for genome sequencing and the ecotype used for the mutagenesis and/or mapping.

Although every mutant requires the design of a custom microarray for enrichment, which could be both costly and take a long turnaround time, we found that microarrays can be stripped and reused several times. Therefore, depending on the size of the genome of interest only a limited set of partially overlapping enrichment arrays could be designed and generated for immediate of-the-shelf usage. For Arabidopsis a set of 10 to 15 microarrays, each targeting about 10 Mb of sequence would probably be sufficient. For larger genomes or regions, it could be considered to first design gene-centric capture arrays, although this strategy potentially ignores part of the underlying biology as certain causal mutations may be missed (e.g. in regulatory regions). Another limitation of enrichment technologies comes with challenges for probe design for capturing in repetitive regions, which as shown in case of the *picup1 *mutant can potentially harbour causative mutation.

Although a successful study has been described identifying a mutation in a bulk segregant population by sequencing and mapping without using prior SNP information in a single experiment, a fairly high and complete genome coverage was necessary [30,31], making the technique challenging, especially for organisms with large genomes.

Finally, the fast forward genetics approach can be applied to any commonly used model system for which complete genome sequences are available. Sequencing requirements do not depend much on the size of a genome as mapping results in the first step largely depend on pool sizes. Candidate loci less than 10 to 20 Mb can typically be expected for any species of interest, which is compatible with the methods described here.

## Conclusions

Taken together, we developed a straightforward two-step approach, termed 'fast forward genetics', which combines bulk segregant techniques with targeted genomic enrichment and next-generation sequencing technology, for the identification of genotype-phenotype relationships. We demonstrate that this method can rapidly prioritize a small number of candidate mutations that can be validated by traditional techniques.

## Methods

### Plant materials, growth conditions, mutagenesis and microscopy

The *twr-1 *allele was generated by EMS mutagenesis as described in [32]. Similarly, the *picup1 *EMS mutant was generated by screening for auxin efflux facilitator polarity defects. Seedlings and embryos were sterilized, plated and grown as described in [33,34]. The Wisconsin insertion mutant WiscDsLox474E07/*twr-2 *(N857510) was obtained from Nottingham Arabidopsis stock center (NASC). Primers for genotyping *twr-2*: p745-LB (Ds-Lox Wisc); AACGTCCGCAATGTGTTATTAAGTTGTC, MTERF-N857510-LP#12; CAAAACCTGGAAAAGATTGAGG, MTERF-N857510-RP#12; GGTCTTGGCATTCCTAATTCC. Aniline blue staining of mature embryos was performed as described in [35].

#### PCR-based mapping and generation of bulk segregant pools

Homozygous *picup1 *or *twr-1 *plants in Columbia-0 (Col-0) background were crossed to Landsberg *erecta *(Ler-1) ecotype to create the mapping populations. For traditional mapping, *twr-1 *and *picup1 *mutants were selected in the F2 generation and DNA was isolated by using a CTAB-based method [36]. Primers for mapping were designed using information from the CEREON collection (http://www.Arabidopsis.org/) and Primer 3 software (http://frodo.wi.mit.edu/).

A pool of 200 seedlings, each with a clear mutant phenotype (homozygous for causative mutation) and a pool of 200 seedlings showing wild-type phenotype (heterozygous for the causative mutation and wild type) were prepared and genomic DNA was isolated with the DNeasy Plant Mini Kit from QIAGEN according to manufacturer's protocol.

#### Preparation of SOLiD libraries

Genomic DNA (1 μg) form the BSA pools was fragmented to ~ 100 nt double-stranded DNA fragments by sonication (Covaris S2, 6 × 16 mm AFA fiber Tube, duty cycle: 20%, intensity: 5, cycles/burst: 200, frequency sweeping, 6 minutes). After fragmentation, fragments were blunt-ended and phosophorylated at 5'-prime end using End-it Kit (Epicentre) according to the manufacturer's instructions. Ligation of double stranded adapters (adapter 1: pre-annealed duplex of 5'-CTA TGG GCA GTC GGT GAT-3' and 5'-ATC ACC GAC TGC CCA TAG TTT-3' and adapter 2: pre-annealed duplex of 5'-CGC CTT GGC CGT ACA GCA G-3' and 5'-GCT GTA CGG CCA AGG CG-3'; all oligonucleotides were acquired through Integrated DNA Technologies (Coralville, IA) and pre-annealing was done by mixing complementary oligonucleotides at 500 μM concentration and running on thermocycler with the following program: 95°C for 3 min, 80°C for 3 min, 70°C for 3 min, 60°C for 3 min, 50°C for 3 min, 40°C for 3 min and 4°C hold), compatible with SOLiD sequencing was performed using Quick ligation kit (New England Biolabs) with 750 mM adaptor 1 and adaptor 2, 150 μl of 2x Quick ligation buffer, 5 μl Quick Ligase in a total volume of 300 μl. Samples were purified using Ampure beads (Agencourt) and amplified in 400 μl of Platinum PCR Supermix with 750 mM of both amplification PCR primers, 2.5 U of Pfu DNA polymerase (Stratagene) and 5 U Taq DNA polymerase (Bioline) (nick translation 72°C for 5 minutes, activation 95°C for 5 min, 8 cycles: 95°C for 15 s, 54°C for 15 s and 70°C for 45 s). After 8 cycles of amplification, library DNA was purified on Ampure beads and size selected on 4% agarose gel for 125-150 bp fraction.

#### Array hybridization and elution

Prior to hybridization 100 ng of stock library was amplified (95°C for 5 min, 10 cycles: 95°C for 15 s, 54°C for 15 s and 70°C for 45 s) in 1000 μl of Platinum PCR Supermix with 750 mM of both amplification PCR primers and 6.25 U of Pfu DNA polymerase (Stratagene) to produce amount of DNA necessary for enrichment (3 μg) and purified using MinElute Reaction Cleanup Kit (Qiagen). Amplified DNA was concentrated by speedvac together with 20× weight excess of salmon sperm DNA and resuspended in 12.3 μl of water. DNA was mixed with 31.7 μl of Nimblegen aCGH hybridization solution and denatured at 95°C for 5 minutes. After denaturing the sample was hybridized for 72 hours at 42°C on MAUI hybridization station. After hybridization, the array was washed using Nimblegen Wash Buffer Kit according the user's guide for aCGH hybridization. Elution was performed using 800 μl of elution buffer (10 mM Tris pH 8.0) in an Agilent Microarray Hybridization Chamber at 95°C for 30 minutes. After 30 minutes the chamber was quickly dissembled and elution buffer was collected. Eluted library DNA was concentrated by speedvac to a volume of 50 μl mixed with 400 μl of Platinum PCR Supermix with 750 mM of both full length amplification PCR primers and 2.5 U of Pfu DNA polymerase (Stratagene) and amplified (activation 95°C for 5 min, 13 cycles: 95°C for 15 s, 54°C for 15 s and 70°C for 45 s).

#### SOLiD Sequencing

We performed deep sequencing of enriched barcoded samples on an AB/SOLiD sequencer (Applied Biosystems) with V3 chemistry according to the manufacturer instructions to produce 50 bp long sequencing reads.

#### Design of enrichment arrays

Capture probes were designed on the repeat-masked sequence of the Arabidopsis reference genome (TAIR8) with a custom PERL script which selects the best 60-mer probe in a local sliding window based on criteria such as melting temperature, mono-nucleotide stretches, GC content. This design window was moved along the sequence with a 2 bp interval, resulting in a dense tiling design of probes. For the reverse strand, a completely independent design was made by offsetting the start point of the first design window. The resulting probe sequences are blasted against the reference genome, and probes with more than 2 hits, with more than 60% match are discarded from the design. The designs were uploaded through Agilent's design website E-Array (https://earray.chem.agilent.com/earray) and produced on a 1 M CGH array. The coordinates for the enriched region are: chr1:21,133,794_22,133,794 (mutant *picup1*, design window slide 2 bp, 347,654 forward, 347,670 reverse probes) and chr5:22,000,600_23,000,600 (mutant *twr-1*, design window slide 2 bp, 426,762 forward, 426,731 reverse probes).

### Analysis of SOLiD sequence data

#### Mapping sequences to the full reference genome

SOLiD sequence tags were mapped to the full genome with the MAQ package [37] (V0.7.1, options: -c, n3, e180, C10) on the Arabidopsis thaliana reference genome (build TAIR8).

#### SNP calling

A custom PERL script was developed to parse the MAQ pileup output and extract SNP genotypes with strict criteria: a minimal coverage of 20, more than 3 unique start sites of reads per allele, variant calls should have a quality higher than 10 and all variant alleles should be called from both strands. A maximum number of identical reads calling the same allele is set to the twice the number of individuals in the pools to suppress clonality effects. Data from both the homozygous and wild type pools is combined in the mapping phase, (creating a virtual F1), mapped and variable positions are determined. These positions are genotyped in the separate pools by either the SNP calling script or a script that checks whether a position is fully reference. Non-reference allele frequencies are calculated in windows of 25 SNPs and plotted.

Raw sequencing data are deposited on GEO archive with accession number: GSE24511. All custom scripts used in this study are available from the authors upon request.

## Competing interests

The authors declare that they have no competing interests.

## Authors' contributions

EC, IJN and MM designed the study. MM, AvD and RB performed the experiments, IJN analyzed the data, IJN, MM, RH, BS and EC contributed to data interpretation and discussion. IJN, MM, and EC wrote the manuscript. All authors approved the final version of manuscript.

## Supplementary Material

Additional file 1 **Figure S1: **A. Simulation of the effect of reducing sequencing coverage depth on mapping results B. Simulation of the effect of the number of SNPs per bin on mapping results. **Figure S2**: Complementation of *picup-1 *mutant with YatY clones. Click here for file
