# Supplementary material for: Identification of factors required for meristem function in Arabidopsis using a novel next generation sequencing fast forward genetics approach
Source: BMC Genomics. 2011 May 20;12:256. doi: 10.1186/1471-2164-12-256 (PMC3114748; doi:10.1186/1471-2164-12-256)

Supplementary Material

Supplementary Figure1:

A. Simulation of the effect of reducing sequencing coverage depth on mapping results

B. Simulation of the effect of the number of SNPs per bin on mapping results.

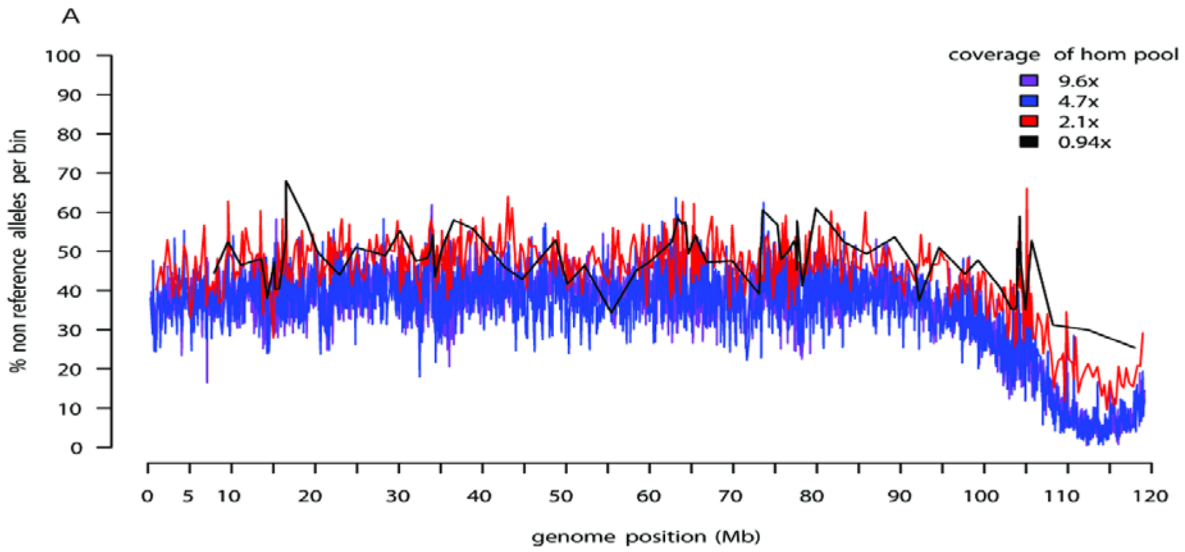

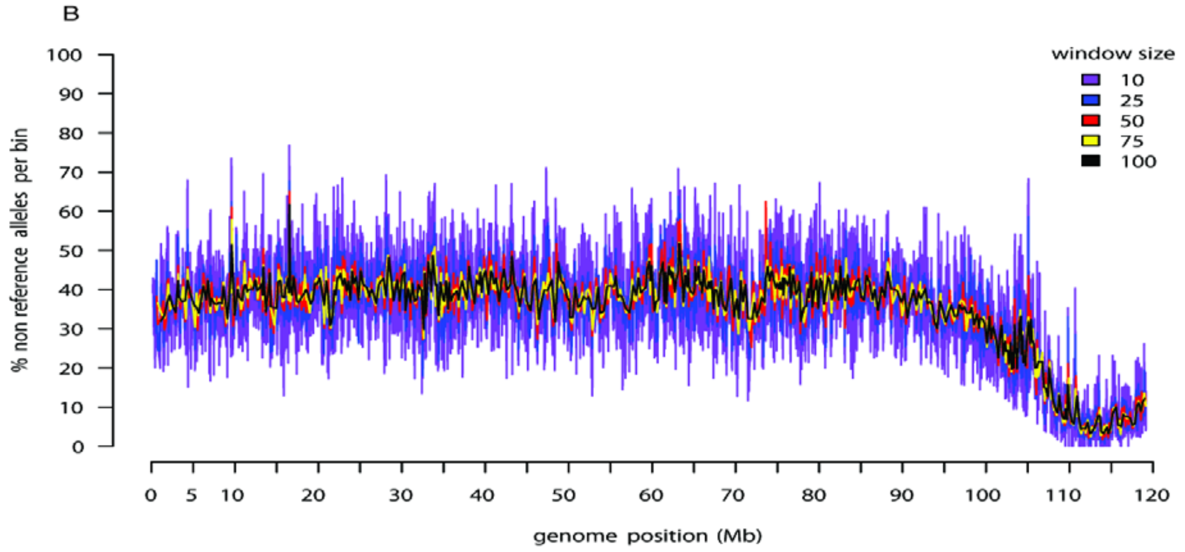

Supplementary Figure2:

Causal variant of *picup*-1 mutant is most likely located in large repetitive region as shown by vertical lines which represent overlapping part of the JatY clones positive in complementation experiments (blue horizontal lines depict 4 clones that did no complement; red horizontal lines depict 4 clones positive in complementation experiments). Non-reference alleles detected in the genomic enrichment sequencing data are plotted by their location and frequency for mutants (black: non-coding, red: coding, blue: UTR). The black boxes indicate repetitive regions.

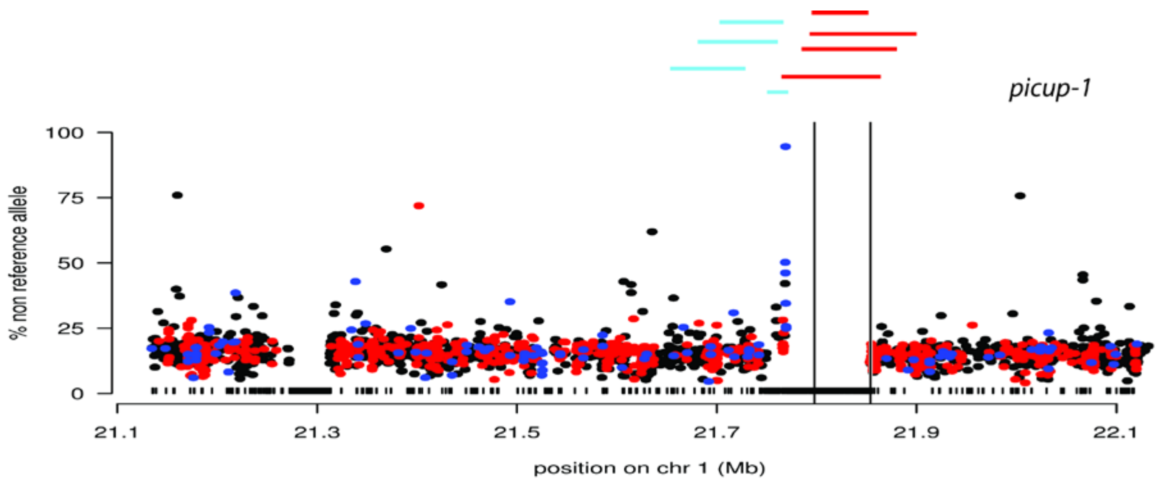

Supplement: Additional file 1 — Figure S1: A. Simulation of the effect of reducing sequencing coverage depth on mapping results B. Simulation of the effect of the number of SNPs per bin on mapping results. Figure S2: Complementation of picup-1 mutant with YatY clones. [file 1471-2164-12-256-S1.PDF]
